# Detection and Prognosis of Prostate Cancer Using Blood-Based Biomarkers

**DOI:** 10.1155/2020/8730608

**Published:** 2020-05-04

**Authors:** Wei Jin, Xiang Fei, Xia Wang, Yan Song, Fangjie Chen

**Affiliations:** ^1^Department of Urology, Shengjing Hospital of China Medical University, Shenyang, Liaoning, China; ^2^Department of Medical Genetics, School of Life Sciences, China Medical University, Shenyang, Liaoning, China

## Abstract

Prostate cancer (PCa) is second only to lung cancer as a cause of death. Clinical assessment of patients and treatment efficiency therefore depend on the disease being diagnosed as early as possible. However, due to issues regarding the use of prostate-specific antigen (PSA) for screening purposes, PCa management is among the most contentious of healthcare matters. PSA screening is problematic primarily because of diagnosis difficulties and the high rate of false-positive biopsies. Novel PCa biomarkers, such as the Prostate Health Index (PHI) and the 4Kscore, have been proposed in recent times to improve PSA prediction accuracy and have shown higher performance by preventing redundant biopsies. The 4Kscore also shows high precision in determining the risk of developing high-grade PCa, whereas elevated PHI levels suggest that the tumor is aggressive. Some evidence also supports the effectiveness of miRNAs as biomarkers for distinguishing PCa from benign prostatic hyperplasia and for assessing the aggressiveness of the disease. A number of miRNAs that possibly act as tumor inhibitors or oncogenes are impaired in PCa. These new biomarkers are comprehensively reviewed in the present study in terms of their potential use in diagnosing and treating PCa.

## 1. Overview

Prostate cancer (PCa), which manifests as solid tumors, is the most prevalent form of cancer in male individuals. PCa is second only to lung neoplasms in terms of mortality rate, and its incidence rises with age in numerous countries. In the US alone, around 2.8 million male individuals are diagnosed annually with PCa [1]. A marked increase has also been noted recently in the incidence of PCa in China due to factors such as lifestyle changes and more efficient diagnosis. The incidence of PCa may also differ due to different dietary habits. Dietary factors, like the consumption of protein, fat, carbohydrates, vitamins, and polyphenols, may have an important role in PCa pathogenesis [2, 3]. Adequate diet and physical activity trigger alterations in the serum factors that retard the development of androgen-reliant PCa cells and cause them to die [2, 4]. Conversely, PCa can be promoted by a high body mass index, hypertension, and a number of metabolic factors [5, 6]. PCa is characterized by a high degree of inhomogeneity, as it can manifest as either latent forms or highly aggressive forms that lead to morbidity and death due to metastasis to other parts of the body [7–9].

PCa screening is based on the use of the prostate-specific antigen (PSA), a feature that has made this disease notably contentious within the healthcare field. PCa exhibited a staggering rise in incidence during the period from 1986–1991, when the PSA test first became available. However, subsequent prostate biopsies frequently revealed no correlation between elevated PSA levels and the presence of cancer in many cases. Indeed, PSA levels do not exceed 4.0 *μ*g/L in 20–25% of the cases with a PCa diagnosis [10]. The results of cytology or histopathology are the basis of a confirmed PCa diagnosis, especially in cases where the PSA levels lie between 4 and 10 *μ*g/L—the so-called “grey area.” By contrast, about one-quarter of the cases with PSA levels of 2–10 *μ*g/L has positive biopsies.

PSA is not an exclusive marker of cancer because it is produced by cancerous prostate cells and by healthy prostate cells. Therefore, factors such as a large prostate size, benign prostatic hyperplasia (BPH), or prostatitis can trigger false positives and result in an incorrect PCa diagnosis and treatment [11]. The United States Preventive Services Task Force issued a recommendation in 2018 that PCa screening should not be performed on the basis of PSA levels in cases of male individuals refusing the procedure or in male individuals 70 years of age or older [12]. Furthermore, unimportant tumors may be excessively detected due to PSA-based screening, leading to unnecessary treatment. In addition, the possible positive outcomes of screening are no greater than the adverse outcomes of excessive detection and unnecessary treatment [13]. Diagnosis of PCa based only on PSA levels is therefore gradually losing its appeal as a clinical approach [14]; however, this has not put an end to the controversy surrounding screening [15, 16]. A recent study suggested that although discontinuation of PSA-based screening would eliminate excessive diagnoses, the lack of screening would lead to a complete failure to avoid preventable deaths and would increase the PCa-related mortality rate by 13-20% [17].

In recent times, various initiatives have been launched to identify novel subtypes of PSA that could make PSA-based screening more precise for diagnosing early PCa and formulating a prognosis for it. These initiatives have yielded several novel PCa biomarkers, including the PHI, a biomarker that has received the approval of the US Food and Drug Administration (FDA) for prostate cancer detection [18]. Another PCa biomarker is the 4Kscore, otherwise known as the 4-kallikrein panel, which screens for total PSA, fPSA, intact PSA (iPSA), and human kallikrein 2. MicroRNAs are another source of promising PCa biomarkers that have emerged as potential alternatives to PSA, owing to the progress being made in deep sequencing technology [19]. Taking all these aspects into account, the aim of the present study is to review the new understanding that has been achieved regarding PCa blood-based biomarkers and the role that they can play in diagnosing and treating early PCa.

## 2. The Prostate Health Index (PHI)

The importance of proPSA as a component of fPSA was highlighted in 1997 by the revelation that the serum of individuals with PCa contained truncated proPSA forms [20]. Preliminary findings confirmed that PCa could be detected based on proPSA isoforms, leading to fewer negative biopsies in cases with PSA levels in the “grey area.” A reliable immunoassay has since been made available on the market by Beckman Coulter to assess [-2] proPSA, and this immunoassay has been extensively investigated for its relevance for use in managing early PCa. Overall, PCa patients have a notably higher ratio of [-2] proPSA to fPSA (%p2PSA).

The [-2] proPSA immunoassay has now been integrated alongside fPSA and tPSA in the PHI test. This test is highly specific for PCa of clinical importance and has proven effective in detecting this disease [21, 22]. It enables differentiation between benign prostatic hyperplasia (BPH) and PCa in suspected cases, but it can also enhance the accuracy of detection of high-grade cancer, thereby preventing redundant biopsies. In June 2012, this biomarker received FDA approval for use in detecting PCa in male individuals 50 years of age or older who had PSA levels of 4–10 *μ*g/L and had undergone a digital rectal examination (DRE) without abnormal outcomes. The National Comprehensive Cancer Network also endorses the PHI test in cases where a biopsy has not been conducted or has produced negative results, and the Network specifies that a significant risk of PCa is reflected by PHI results higher than 35 [23].

Prospective research conducted in multiple centers among a sample of 2499 men has indicated that the use of PHI values higher than 30 at 90% sensitivity for high-grade PCa (HGPC) (Gleason ≥ 7) could have prevented Gleason 6 PCa diagnoses in 33% of the cases and biopsies in 56% of the cases of Asian men. Similarly, the use of PHI higher than 40 at 90% HGPC sensitivity could have prevented Gleason 6 PCa diagnoses in 31% of the cases and biopsies in 40% of the cases of Caucasian men [24, 25]. Prediction of biopsy results based on PHI and the %p2PSA score has now been advocated by a number of researchers. The usefulness of these measurements in cases where the PSA levels fall between 2 and 10 *μ*g/L has been demonstrated by two studies conducted in multiple centers in 1362 and 646 patients. The %p2PSA was associated with an area under the curve (AUC) value of 0.72, while the PHI was associated with an AUC value of 0.74 [23]. Both biomarkers considered together were associated with an AUC value of 0.67 [26]. Chiu et al. suggested that PHI was effective in cancer risk stratification for both European and Asian subjects [25]. However, population-specific PHI reference ranges were different and should be used [25]. These findings support the relevance of the PHI test for identifying cases among Asian and Caucasian individuals with a high risk of PCa and thereby minimizing redundant biopsies and excessive PCa diagnosis.

When compared with the total PSA and %fPSA, the combination of %p2PSA and PHI provided a greater precision of PCa detection at biopsy [23, 27]. Furthermore, the two biomarkers were well-correlated with tumor aggressiveness, occurring at higher levels in cases of Gleason > 6. These findings have been confirmed by two meta-analyses from 2013 and 2014, which indicated that %p2PSA and PHI were, respectively, associated with AUCs of 0.635–0.78 and 0.69–0.781 [28, 29]. These meta-analyses additionally highlighted the superior outcomes obtained with these two biomarkers compared with those obtained with PSA and %fPSA. The latter observation was reinforced by a later meta-analysis, which undertook a comparison between PHI and %fPSA in cases with total PSA levels of 2–10 *μ*g/L. In that meta-analysis, PHI had an AUC value of 0.74, while %fPSA had an AUC value of 0.63 [30].

Some researchers have argued that PCa could be predicted clinically with greater accuracy by incorporating PHI and %p2PSA into multivariate models that take into account the integration of PSA and a range of clinical and demographic factors. For instance, the incorporation of PHI and %2pPSA into a multivariate model based on patient age, prostate volume, PSA, and %fPSA was reported to increase the AUC value from 0.762 to 0.802 or 0.815, depending, respectively, on whether a logistic regression analysis or an artificial neural network was applied [31]. Similarly, a different study on 112 patients with Gleason 6 who underwent radical prostatectomy reported that the patients with a final histology upgrade to Gleason 7 or higher exhibited markedly increased PHI values [32]. Another study found that PHI had an AUC value of 0.815 for the detection of GHPC in cases of Gleason 7 or higher [33] and suggested that the ideal PHI cut-off point of 24 at 95% sensitivity for detection of aggressive PCa. The use of this cut-off point prevented redundant biopsies in 41% of the cases. By contrast, an active monitoring program involving 167 patients revealed that the baseline and longitudinal %p2PSA and PHI levels allowed better prediction of renewed biopsy categorization at follow-up, whereas total PSA level showed no similar correlation with renewed biopsy categorization [34].

## 3. The 4Kscore

Another test that can predict the likelihood of occurrence of high-risk PCa is the 4Kscore or 4-kallikrein panel. The 4Kscore consists of four kallikrein blood markers—total PSA, free PSA (fPSA), intact PSA (iPSA), and the human kallikrein-related peptide 2 (hK2)—and has received ample research attention. For example, researchers from the Memorial Sloan-Kettering Cancer Center have found an association between the 4Kscore and AUC values that exceeded not only the AUC values associated with a PSA-based model for the detection of all types of PCa (AUCs = 0.674–0.832) but also the AUC values for HGPC detection with Gleason of 7 or higher (AUCs = 0.793–0.870) [35, 36]. A comparison of the equivalent clinical models for high-risk PCa prediction yielded similar results. The model incorporating the variables of PSA, patient age, and DRE had AUC values of 0.709–0.868, whereas the addition of fPSA, iPSA, and hK2 to the model led to AUC values of 0.798–0.903 [37].

One major factor that PCa risk predictions typically take into account is prostate volume, as this factor has an impact on the levels of PSA in the serum. However, one study reported that the method used to perform the 4Kscore to detect PCa was unaffected by the prostate volume [38]. This was the conclusion reached from a biomarker investigation in two groups of patients (*n* = 2914, *n* = 740, respectively) with PSA levels of 3 *μ*g/L or higher. The inclusion of the prostate volume in a model underpinned by 4Kscore, patient age, and DRE led to an increase in AUC from 0.856 to 0.860 in the first group, whereas the inclusion or exclusion of the prostate volume made no difference to the AUC value in the second group (0.802).

The 4Kscore was made available on the market by Opko Diagnostics. It constitutes an algorithm that generates predictions about HGPC by integrating the 4-kallikrein panel with patient age, DRE, and history of previous biopsy. The US Food and Drug Administration FDA has not yet approved this test, but the National Comprehensive Cancer Network issued a recommendation in June 2015 that the 4Kscore could be employed to screen for HGPC in cases where biopsy had either not been conducted or where it had produced negative results [39, 40].

In a prospective study conducted in 26 urology centers throughout the US, the 4Kscore achieved an AUC value of 0.82 when it was used to assess 1012 male patients about to undergo a prostate biopsy. The use of a 6% cut-off point was found to eliminate the necessity of a biopsy in 30% of the cases and deferred diagnosis in only 1.3% of the HGPC cases [41]. By contrast, a different study reported that the 4Kscore enabled the prediction of HGPC in cases with PSA levels exceeding 10 *μ*g/L or with a positive DRE [42]. This ability of the 4Kscore to decrease the number of biopsies without overlooking a significant proportion of HGPCs has been highlighted by a number of studies [43, 44]. The biopsy was deemed unnecessary in approximately half of the 262 cases scheduled for prostate biopsy because of PSA levels of 3 *μ*g/L or higher, but the diagnosis of aggressive PCa was overlooked in just 1% of these cases when the 4Kscore was used [45]. A different study on a sample of 740 male individuals who had been referred for biopsy due to high PSA levels reported comparable results [46].

The efficacy of the 4Kscore for predictions was demonstrated by a study initiated in 1986 in Sweden on a sample of patients of different ages (40, 50, and 60 years old) [47]. The patients were divided into two groups based on their 4Kscore outcomes at 50 and 60 years of age to determine how likely they were to develop remote metastasis after two decades. The study concluded that remote metastasis was more likely to develop in patients with a 4Kscore exceeding 5 at 50 years of age and with PSA levels of 2 *μ*g/L or higher, as well as in patients with a 4Kscore exceeding 7.5 at 60 years of age and PSA levels of 3 *μ*g/L or higher. Biopsy was considered unnecessary in cases that had moderate increases in PSA levels during midlife and a low risk of HGPC based on the 4Kscore. A different study on 1423 PCa patients showed that remote metastasis occurred in 235 patients with PSA levels exceeding 2 *μ*g/L. However, metastasis was much better predicted by a prespecified model underpinned by the four kallikrein markers than by PSA alone [47]. On the whole, the available research results support the efficiency of the 4Kscore for use in early PCa diagnosis and prognosis.

One study compared the efficacy of the 4Kscore and PHI in a sample of 531 male individuals with PSA levels of 3–15 *μ*g/L. Neither biomarker differed markedly in the detection of either any-grade PCa or HGPC. The 4Kscore had AUC values of 0.69 and 0.718, while the PHI had AUC values of 0.704 and 0.711 [48].

## 4. The Usefulness of MicroRNAs as PCa Biomarkers

### 4.1. miRNAs and PCa

Ample research has recently explored the function of protein-coding genes in oncogenesis. Transcription of the human genome to messenger RNA (mRNA) occurs in a proportion of 90%, but protein encoding is undertaken by just 2% of the genome. Therefore, many RNAs do not encode for any protein. According to the findings from the latest studies, a genome segment without protein encoding is involved in oncogenesis [49, 50].

The expression of genes is regulated by endogenously expressed small, noncoding, single-stranded RNAs known as microRNA (miRNAs). Adverse regulation of gene expression by miRNA genes occurs after transcription, with the miRNA genes attaching primarily to the 3′ untranslated region (3′-UTR) of the coding gene, thereby suppressing mRNA translation. In the case of PCa, this suppression also promotes pathways for PCa progression by triggering translational repression or mRNA deterioration [49, 50]. The types of miRNA complexes present in human plasma and serum were investigated in one study by differential centrifugation and size-exclusion chromatography of the miRNAs [51]. Most of the miRNAs were found in the plasma and serum in a form that did not undergo membrane binding, whereas a few were vesicle-associated miRNAs. A different study suggested that miRNAs from human serum, saliva, and other biological fluids could be made more sensitive through exosome isolation [52].

The physiological and pathological processes showing miRNA involvement include development, differentiation, metabolism, immunity, proliferation, apoptosis, senescence, cell identity, and stem cell maintenance [50, 53]. A number of miRNAs that participate in the development of human cancer have been identified, and these appear to act as oncogenic factors that prevent the activity of tumor-suppressing genes or as tumor-suppressing miRNAs that activate oncogenes [54].

Novel biomarkers for the diagnosis and prognosis of cancer can be discovered by investigating cancer-related abnormal miRNA expression profiles. Many methods have been used to detect miRNAs, including northern blotting, next-generation sequencing (or RNA sequencing, RNA-Seq), microarrays analysis, reverse transcription PCR (RT-PCR), highly sensitive biosensors, and computational prediction [55]. In 2008, the first circulating miRNA profile of the serum of patients with diffuse large B-cell lymphoma was published as an aid for cancer diagnosis [56]. In many human cancers, the processes of development, invasion, and progression occur through direct involvement of hundreds of miRNAs with modified expression, and the cancer-related genomic area contains approximately half of the miRNA genes. PCa development is promoted by this type of dysfunctional regulation through the upregulation of oncogene modulation or downregulation of tumor inhibitors [50].

The current findings indicate the notable feature that the classification of human cancer origins can be based on miRNA signatures [57]. Thus, metastatic cancers of unknown origin can be effectively identified with high tissue specificity based on their miRNAs. For instance, a study of 208 tumors consisting of 15 distinct histologies, including PCa, revealed successfully and estimated the primary tumor origin by profiling miRNA expression in paraffin-embedded tissue [58]. A novel algorithm formulated in that study also categorized the miRNAs with 85% general precision (CI: 79–89%). This algorithm was successful in determining the primary origin of 42 of 48 metastases (88% precision; CI: 75–94%). However, these results must be verified by further research.

Several studies have highlighted an association between PCa and the modifications observed in the expression of miRNAs, which are associated with dysfunctional cell proliferation, differentiation, progression, and other processes [59, 60]. Comprehending the mechanism by which miRNAs interact with their targets and the implications of those interactions for PCa development is important for identifying the central part played by miRNAs in PCa [61]. This comprehension can also help in developing efficient treatment interventions, especially because aberrant miRNA expression is now viewed as a useful biomarker for diagnosing and classifying PCa and for determining its prognosis [62].

### 4.2. Oncogenic miRNAs and PCa

Cancer cell progression is promoted by miRNAs that serve as oncogenic miRNAs [63]. Calin and colleagues [64] were the first to investigate the correlation between miRNA dysregulation and cancer. Specifically, the authors observed that the dysregulation of miR-15 and miR-16 contributed to the development of chronic lymphocytic leukemia. PCa is typically associated with overexpression of miRNAs (e.g., miR-21, miR-32, miR-221, miR-222, miR-181, miR-18a, and miR-429) that have a crucial function in the regulation of PI3K/AKT, the epithelial-to-mesenchymal transition (EMT), cell proliferation, apoptosis, and androgen receptor (AR) expression [65, 66].

The androgen-regulated oncogenic miRNAs with expression in PCa include miR-21 and miR-32 [67]. MiR-21 regulates the expression of a large number of mRNA targets associated with microvascular proliferation and tumor invasiveness, and its expression is correlated with weak biochemical recurrence-free survival. Therefore, miR-21 expression can be used to estimate the probability of biochemical recurrence in PCa patients who have undergone radical prostatectomy [68]. The expression of miR-21 is also associated with castration resistance and metastasis, and its level increases linearly with clinical parameters such as the Gleason score and lymph node metastasis [59]. A study conducted on patients who had undergone radical prostatectomy revealed a close correlation between elevated miR-21 levels and advanced disease staging, lymph node metastasis, and poor patient outcomes. A multivariate analysis revealed that miR-21 expression was an independent estimator of biochemical recurrence after five years [69]. Hence, miR-21 can be used as a biomarker that anticipates cancer progression [70].

An additional miRNA with androgen regulation (via dihydrotestosterone [DHT] stimulation) is miR-32. This miRNA has a higher expression in castration-resistant prostate cancer (CRPC) cases than in benign prostatic cases. Its oncogenic activity includes targeting B-cell translocation gene 2 (BTG-2) as well as phosphoinositide-3-kinase interacting protein 1 (PIK3IP1). This miRNA suppresses apoptosis and encourages cells to proliferate by stimulating cell growth, as revealed by observations of miR-32 effects in the LNCaP PCa cell line. On the whole, miR-32 manifests its oncogenic activity by targeting the tumor-suppressing genes that control the ability of cells to proliferate, survive, and migrate [71, 72].

The oncogenic reprogramming of PCa-mobilized stem cells is believed to occur with the involvement of oncogenic miR-125b, miR-130b, and miR-155 [73, 74]. Mechanistic studies confirm that stem cell neoplastic reprogramming is likely triggered by miR-125b and miR-155 exosomal trafficking via targeted suppression of the sizable tumor-suppressor homolog2 and programmed cell death by the neoplastic transformation suppressor protein 4 [75]. Furthermore, miR-125 has been observed to target apoptosis-promoting genes in PCa, thus functioning as an oncogene [76]. Apoptosis is suppressed and cell proliferation is intensified when miR-125b, a miRNA triggered by an androgen receptor, is activated in the LNCaP cells. Some evidence supports miR-125b targeting of the mediator of Mdm2 sequestration, p14^ARF^ and subsequent disruption of Mdm2 deterioration, and triggering of the p53 network [77].

The expression of p27^Kip1^, which regulates the cell cycle, is susceptible to the effects of several miRNAs, such as miR-221, miR-222, and miR-429 [78]. Two of these, miR-221 and miR-222, are encoded on the X chromosome and share a seed sequence. Both of these miRNAs manifest their oncogenic effects by targeting a number of tumor-suppressing genes, and they both display overexpression in PCa. Abnormal expression of these two miRNAs can aid in prognosis because this expression has a close association with metastatic CRPC [78].

A modified miR-9 signature has been correlated with the progression and metastasis of PCa [79]. Similarly, miR-27a targets prohibition in PCa, thereby regulating AR processing [61]. PCa tissues and cell lines also show high expression of miR-18, which targets the tumor-suppressing serine/threonine-protein kinase 4, thereby functioning as an oncogenic miRNA [80]. One study showed that the inhibition of miR-18a slowed PCa cell and tumor growth by promoting apoptosis triggered by Akt dephosphorylation with STK4 mediation [80]. These findings support the use of the peripheral blood oncogenic miR-18a as a noninvasive PCa biomarker and for differentiating PCa from BPH [81].

Important stages in PCa progression are associated with poor prognosis; these include bone metastasis and events affecting the skeleton [82]. Extensive research has been conducted on the involvement of miRNAs in cancer metastasis. PCa has been associated with the upregulation of miR-96, miR-154, and miR-409-5p, which are likely very important promoters of cancer colonization of bone during metastasis [83–86]. Additional evidence produced by targeting major fibroblast-activating molecules has shown a correlation between PCa progression and increased expression of miR-133b, miR-409, and miR-210 [84, 85].

### 4.3. Tumor-Suppressing miRNAs and PCa

Tumor-suppressing gene action may be an additional function of miRNAs. Cancer cells generally exhibit downregulation of tumor-suppressing miRNAs, thereby promoting proliferation [87], the epithelial-mesenchymal transition (EMT), invasiveness, and metastasis. PCa progression could be monitored by evaluating the expression pattern of tumor-suppressing miRNA as a biomarker, and the same pattern could also be targeted by various treatment interventions [88].

Reduced expression of miRNA has been associated with cancer progression in a number of studies. PCa with castration resistance can develop through priming of AR and AR-V7 upregulation with hnRNPH1 mediation following dysregulation of miR-212 [89]. The PCa tumor promoter EGFR is targeted by miR-133 and miR-146a, thereby preventing disease progression. Therefore, intensified EGFR signaling may explain the loss of these two biomarkers and the subsequent aggressive progression of PCa [90]. Evidence has also been produced to indicate that the LNCaP, DU145, and PC3 PCa cell lines, as well as PCa tissues, exhibit reduced expression of miR-335 [91]. Another miRNA that inhibits tumors is miRNA-30a, a miRNA that targets sine oculis homeobox homolog 1 to prevent cancerous cells from proliferating and invading other tissues [92].

PCa is also associated with the downregulation of miR-145, miR-200, miR-29b, miR-205, and miR-940, which leads to the upregulation of genes associated with the EMT. During the EMT, cells lose their attachment to other cells, and epithelial cells acquire the abilities of migration and invasion through conversion to mesenchymal stem cells capable of differentiation into various other cell types. Reduced expression of miR-29b and miR-130b in PCa is also correlated with extracellular matrix regulation through the targeting of matrix metalloproteinase 2 (MMP2) [93, 94]. EMT and cancer invasion are inhibited by miR-200 members through a direct suppression of the zinc-finger E-box binding homeobox 1 and 2 (ZEB1 and ZEB2) translation factors [95]. Both prostate tumors and cancer cell lines have been associated with miR-200b downregulation, and miR-200b expression contributes significantly to the regulation of tumor invasion, metastasis, and chemosensitivity [96]. Enhanced expression of miR-200b targets Bmi-1, thereby preventing the proliferation and migration of PCa cells, as well as increasing PCa cell chemosensitivity to docetaxel.

In general, hindering the EMT by miR-200c appears to support the maintenance of the “epithelialness” of cancer cells [97, 98]. According to one study, PCa was prevented from progressing by miR-205 downregulation that targets laminin-332, integrin-*β*4, and MMP-2 to regulate the extracellular matrix [99]. In addition, miR-205 targets the Bcl-2 protein to suppress apoptosis. The loss of miR-205 is significant in androgen-autonomous expression equivalent to Bcl-2 upregulation and possibly in reduced sensitivity to docetaxel in PCa [100].

An association has been established between decreased expression of miR-146a in PCa and angiogenesis suppression through targeting of the epidermal growth factor receptor pathway [101]. The progression and metastasis of PCa are stimulated by miR-1 downregulation through targeting of Src and TWIST1 [102]. EMT regulation and PCa progression are also mediated by targeted downregulation through the loss of miR-154 due to stromal antigen 2 (STAG2), the loss of miR-203 due to tumor growth factor-alpha (TGF-*α*), and the loss of miR-224 due to Tribbles Pseudokinase 1 (TRIB1) [103, 104].

The original members of the family of tumor-suppressing miRNAs are miR-15a and miR-16. These two miRNAs are in charge of regulating the expression of numerous oncogenes, such as BCL2, MCL1, CCND1, and WNT3A. The effects of miR-15 and miR-16-1 on the BCL2 gene product trigger apoptosis [105–107]. By contrast, the loss of these two miRNAs elevates the levels of cyclin D1, an important regulator of the transition from the G1 to the S phase. The loss of these two miRNAs also activates the WNT3a gene and the precancerous Wnt signaling pathway. Notably, cancer progression is stimulated by the cooperation between increased miR-21 expression and the loss of miR-15 and miR-16, as revealed by mouse models of PCa. One study found that some PCa patients exhibited deregulated miR-15/miR-16 and miR-21, and this deregulation was related to suboptimal prognosis and disease-free survival [108].

The expression and activity of the AR gene also seem to be targeted by a number of tumor-suppressing miRNAs. Proliferation is inhibited and apoptosis intensified by miR-488∗ [109], which can successfully reduce the expression of the AR protein in androgen-responsive as well as androgen-refractory PCa cells. PCa cells that overexpress miR-488∗ have also been shown to have lower PSA expression. Similarly, the direct downregulation of AR expression by excessive expression of miR-488∗ in PCa cells can prevent cell growth and enhance apoptosis [109].

AR expression is significantly affected by miRNAs like miR-125b, miR-135a, and miR-27a [76, 110]. Androgen-responsive PCa cells display the upregulation of miR-135a, which therefore seems to have a tumor-inhibiting effect. An androgen-responsive component is also demonstrated by miR-125b in the gene promoter area. Research on castrated mice revealed that the upregulation of miR-125b caused prostate tumor xenografts to exhibit androgen-independent growth, suggesting that the miRNA suppressed apoptosis [110].

AR expression regulates the expression of miR-27a as well. The expression of prohibitin, a tumor-suppressing gene, and AR corepressor, is downregulated when miR-27a expression is increased through androgen mediation. By facilitating a reduction in the expression of the prohibitin protein product, miR-27a upregulates the AR target gene expression and enhances the growth of PCa cells [111]. On the whole, the wide range of functions fulfilled by miRNAs in PCa development has received substantial attention in the search to understand how miRNAs aid and inhibit the disease process.

### 4.4. Exosomal miRNAs

The shortcomings of the existing protocols for PCa screening call for the discovery of novel biomarkers. Effective management of this disease and improvements in prognosis and survival can only come with protocols that will allow the detection and classification of prostate tumors as early as possible. Another potential source of biomarkers is exosomes. These nanovesicles measure 50–150 nm and are found in cells as early endosomes that contain miRNAs, mRNA, proteins, and lipids. Exosomes play a key role in intercellular communication and in the regulation of recipient cell biology, as well as in a range of physiological and pathological processes [112]. Some evidence now supports the idea that miRNA can be reliably sourced from blood-derived exosomes to identify disease biomarkers [113, 114].

In numerous tumor types, prognosis has been predicted based on exosomal miRNAs recovered from the blood [115]. The relevance of using the miRNAs found in exosomes to manage PCa was investigated in a few studies. Two of these studies have suggested that PCa could be differentiated based on miR-141 upregulation [116, 117]. Another study reported that PCa was associated with the upregulation of both miR-141 and miR-375 and that disease progression was correlated with the serum levels of these miRNAs [118]. A correlation has also been established between poor survival outcomes and plasma levels of exosomal miR-1290 and miR-375. Better predictions were achieved when these novel biomarkers were included in a clinical prognostic model [119, 120]. miR-574-3p, miR-141-5p, and miR-21-5p were markedly upregulated in urinary exosomes from PCa patients extracted via lectin-based exosome agglutination [121]. By contrast, extraction of urinary exosomes by differential centrifugation revealed a marked increase only in the expression of miR-141. The presence of miR-19b was also examined in urinary exosomes extracted via differential centrifugation and revealed good potential for PCa detection [122].

Despite the inconsistencies in the reported results, the initial findings regarding exosomal biomarkers are encouraging. However, the determination of the usefulness of exosomes will first require standardization of the isolation and profiling methods and the performance of extensive clinical trials. Furthermore, miRNAs are not without their shortcomings and difficulties, just like other novel biomarkers for cancer. These shortcomings and difficulties stem from differences in how samples are selected, handled, processed, and prepared, as well as from disagreements regarding data normalization. Consequently, before miRNAs can be employed clinically, those shortcomings and difficulties must be addressed.

## 5. Conclusion

A great deal of inhomogeneity occurs in PCa. In some cases, the risk of disease progression is minimal, and the cancer-specific survival rates at a 15-year follow-up can be higher than 99%. Around 50–60% of new diagnoses are low-risk PCa [123]. However, aggressive PCa is associated with a significant reduction in survival rate. Therefore, detection of the disease as early as possible is vital, as is the effective treatment of these cases. These advances can only come about with the development of better methods of early diagnosis to improve the survival rate.

A number of novel biomarkers related to PCa aggressiveness have been recently shown to have great clinical potential. The outcomes of prospective studies conducted in multiple centers have indicated that the use of the PHI and 4Kscore biomarkers decrease the proportion of redundant biopsies in male individuals subjected to PSA screening. Various guidelines have advocated the use of these biomarkers [124, 125]. Nevertheless, comparative analysis of the usefulness of biomarkers requires large-scale prospective studies and stringent control of the bias caused by patient preselection based on the levels of PSA in the serum.

Several miRNAs with possible oncogene or tumor-suppressing functions are deregulated in PCa. As revealed by miRNA profiling research, mRNAs can regulate gene transcription either on their own or in conjunction with other transcription factors, with the end result being disrupted cellular processes in PCa tissues [18, 125]. Novel approaches to PCa management could be identified by advances in the knowledge of the functions of miRNAs in controlling PCa development and progression [126, 127]. Research on the integrated function of biomarkers and combination with magnetic resonance imaging data is also worthwhile [128, 129]. Comparative analyses are also needed on the outcomes achieved with blood biomarkers and the encouraging data yielded using PSA or PHI. It should be known that none single biomarker has the capacity to perfectly detect PCa, and more detailed profiling of novel PCa biomarkers remains necessary.
